# Structural insights into the membrane-extracted dimeric form of the ATPase TraB from the *Escherichia coli *pKM101 conjugation system

**DOI:** 10.1186/1472-6807-11-4

**Published:** 2011-01-25

**Authors:** Eric Durand, Gabriel Waksman, Veronique Receveur-Brechot

**Affiliations:** 1 Institute of Structural and Molecular Biology, UCL/Birkbeck, Malet Street, London WC1E 7HX, UK; 2 IMR-CNRS - UPR3243, 31 Chemin Joseph Aiguier, 13402 Marseille Cedex 20, France; 3 LISM-CNRS - UPR9027, 31 Chemin Joseph Aiguier, 13402 Marseille Cedex 20, France

## Abstract

**Background:**

Type IV secretion (T4S) systems are involved in secretion of virulence factors such as toxins or transforming molecules, or bacterial conjugation. T4S systems are composed of 12 proteins named VirB1-B11 and VirD4. Among them, three ATPases are involved in the assembly of the T4S system and/or provide energy for substrate transfer, VirB4, VirB11 and VirD4. The X-ray crystal structures of VirB11 and VirD4 have already been solved but VirB4 has proven to be reluctant to any structural investigation so far.

**Results:**

Here, we have used small-angle X-ray scattering to obtain the first structural models for the membrane-extracted, dimeric form of the TraB protein, the VirB4 homolog encoded by the *E. coli *pKM101 plasmid, and for the monomeric soluble form of the LvhB4 protein, the VirB4 homolog of the T4S system encoded by the *Legionella pneumophila lvh *operon. We have obtained the low resolution structures of the full-length TraB and of its N- and C-terminal halves. From these SAXS models, we derive the internal organisation of TraB. We also show that the two TraB N- and C-terminal domains are independently involved in the dimerisation of the full-length protein.

**Conclusions:**

These models provide the first structural insights into the architecture of VirB4 proteins. In particular, our results highlight the modular arrangement and functional relevance of the dimeric-membrane-bound form of TraB.

## Background

Type IV secretion (T4S) systems are one of six secretion systems used to transport effector proteins or DNAs through the cell membrane of Gram-negative bacteria. These six secretions systems can be categorised into two classes. The first class of secretion systems mediates substrate transfer from the cytosol to the extracellular milieu in one step: substrates captured from the cytosol are released extracellularly without the need for a periplasmic intermediate [1]. The second class encompasses a range of specialised outer membrane (OM) secretion systems: the substrate is first transported through the inner membrane (IM) to the periplasm via the general SecABYEG secretion machinery and then uses specialised OM systems for extracellular release [2,3]. T4S systems belong to the first class.

T4S systems export proteins and DNA-protein complexes and fulfil a wide variety of functions, such as i- the conjugative transfer of plasmids and other mobile DNA elements to bacterial recipient cells, ii- the direct uptake of DNA from the extracellular milieu or iii- the delivery of protein or DNA substrates to eukaryotic target cells [4,5]. T4S systems are used by several plant and human pathogens for virulence. Such bacterial pathogens include *Agrobacterium tumefaciens*, the causative agent of crown gall disease in plants, *Bordetella pertussis*, the agent responsible for whooping cough in children, and *Helicobacter pylori*, responsible for gastric ulcers and stomach cancer [6-9]. In addition, there are intracellular bacterial pathogens utilising T4S systems for virulence, such as *Brucella suis*, the causative agent of brucellosis, and *Legionella pneumophila*, the causative agent of Legionnaires' disease [10,11].

T4S systems are generally composed of 12 protein components forming a macromolecular assembly inserted into the bacterial cell envelope [5]. These proteins are named VirB1-VirB11 and VirD4, based on the widely used nomenclature of the model system, the *A. tumefaciens *VirB/D4 T4S system. Three ATPases are key components of the T4S system: VirD4, VirB11 and VirB4. VirB4 proteins are the largest and the most evolutionarily conserved proteins in T4S systems [12] but their function remains unclear. Although VirB4 proteins have clearly defined Walker A and Walker B motifs characteristic of ATPases [13], until very recently no ATPase activity had been demonstrated for any VirB4 homologues [13]. However, two recent studies have shown that ATPase activities of VirB4 proteins are crucially dependent on solution conditions and on the oligomerisation state of VirB4 [14,15]. For TrwK, the VirB4 homolog encoded by the R388 conjugative plasmid system, Rabel *et al. *[13] initially reported that the protein exhibited no ATPase activity and was monomeric. However Arechaga et al. [14], in a subsequent study, reported an ATPase activity of TrwK in the presence of acetate ions, possibly due to a small proportion of an hexameric form of the protein. TraB, the VirB4 homolog encoded by the pKM101 conjugative plasmid system, also exhibits ATPase activity in the presence of acetate ions and is primarily hexameric under these solution conditions [15]. Interestingly, TraB partitions between the cytosol and the inner membrane, and the membrane-extracted form does not exhibit ATPase activity, even in the presence of acetate ions [15]. This membrane-extracted form of TraB was also shown to be dimeric. It was concluded that cytosolic TraB is in equilibrium between a dimeric form that binds DNA and nucleotides, but is unable to hydrolyze ATP, and an acetate-induced hexameric form able to hydrolyse ATP. TraB purified from the membrane is in the dimeric form, and is unable to transition to the hexameric form even in the presence of acetate ions [15]. Interestingly, *A. tumefaciens *VirB4 was also shown to form active dimers *in vivo *[16], strongly supporting a functional role of this dimer, besides the hexameric form.

The structure of VirB4 proteins is still unknown, as they have resisted extensive crystallisation efforts either in the hexameric or the dimeric form. Attempts at visualising acetate-induced hexameric TraB by negative stained electron microscopy or small-angle X-ray scattering have failed [15]. Recently, based on sequence similarities with TrwB (the VirD4 homolog from the plasmid R388 conjugation system), the *A. tumefaciens *VirB4 C-terminal domain was modelled, as an homo-hexameric ring [17] much like VirB11 and VirD4 [18]. However no structural experimental data has yet backed this model, most probably because it has been impossible so far to stabilise and isolate the hexameric form of VirB4. Here we report the low resolution structure of the membrane-extracted dimeric form of TraB, using small-angle X-ray scattering (SAXS). We also performed a SAXS analysis of the N-terminal (TraB_NT_) and C-terminal (TraB_CT_) domains of TraB, and of the full-length monomeric LvhB4, the VirB4 homolog from the *L. pneumophila *T4S system, which represents the first *in vitro *study of a member of the *L. pneumophila lvh *T4S system. Altogether, our results provide the first insights into the architecture of the highly conserved VirB4 family of proteins.

## Results

### Purification of TraB domains and LvhB4

**Figure 1:**
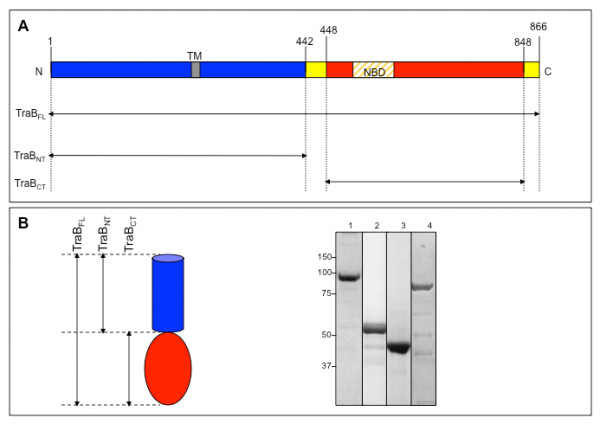
**Schematic representation of the domain structures of TraB**. (A) N: N-terminus; C: C-terminus; TM: Putative transmembrane domain; NBD: NTP binding domain; FL: TraB full-length (TraB_FL_: 1-866); NT: TraB N-terminal domain (TraB_NT_: 1-442); CT: TraB C-terminal domain (TraB_CT_: 448-848). (B) NuPAGE 4-12% showing the purified proteins after gel filtration. (Lane 1) TraB_FL _(102 kDa); (Lane 2) TraB_NT _(55 kDa); (Lane 3) TraB_CT _(48.8 kDa); and (Lane 4) LvhB4 (93.8 kDa). Molecular mass markers are indicated on the left side of the gel (kDa). A transmembrane segment (TM) in TraB is predicted, between residues 254 and 271.

Based on sequence homology between the C-terminal domain of TraB and the protein TrwB, the *E. coli *R388-encoded VirD4 homolog, we previously established that TraB can be divided into two folded domains (Figure 1A): a C-terminal domain (residues 448 to 848, TraB_CT_), which is soluble, and an N-terminal domain (residues 1-442, TraB_NT_), which partitions between a soluble form in the cytosol and a membrane-bound form [15]. These two domains are functional as they are each able to bind DNA and ATP [15]. Full-length TraB (TraB_FL_) was subsequently found to partition between a soluble and membrane-bound form [15]. Various transmembrane domains predictors were used to screen the sequence of TraB (DAS, http://www.sbc.su.se/~miklos/DAS/; HMMTOP, http://www.enzim.hu/hmmtop/; TMPred, http://www.ch.embnet.org/software/TMPRED_form.html; TMHMM, http://www.cbs.dtu.dk/services/TMHMM-2.0/; and TopPred, http://mobyle.pasteur.fr/cgi-bin/portal.py?form=toppred). The predictions were not fully consistent, except for one stretch that was predicted by two out of the 5 different predictors, between residues 254 and 271. This suggests the existence of a transmembrane (TM) segment in TraB, or of a hydrophobic patch through which TraB might be associated with the membrane.

For this study, TraB_FL _and TraB_NT _were both purified from the membrane fraction, while TraB_CT_, which is soluble and does not partition in the membrane, was purified from the soluble fraction. For comparison, we cloned, expressed and purified the full-length LvhB4, the VirB4 homolog from the *L. pneumophila *T4S system, for which no predicted TM domain was found. Indeed, LvhB4 purifies from the soluble fraction and not from the membrane fraction, demonstrating that the protein is not located in the membrane. All four proteins were purified to homogeneity using the same two-step purification strategy (Figure 1B and "Materials & Methods"). In SDS-PAGE the proteins migrate at their expected molecular mass: 102 kDa for TraB_FL_, 55 kDa for TraB_NT_, 49 kDa for TraB_CT_, and 94 kDa for LvhB4 (Figure 1B).

### Size Determination of TraB domains and LvhB4

We investigated the oligomeric state of all four proteins in the GF^sol ^(TraB_CT _and LvhB4) and GF^mb ^(TraB_NT _and TraB_FL_) buffer conditions (see definition of GF^sol ^and GF^mb ^in Materials and Methods). Table 1 summarizes the results obtained by Gel Filtration, Dynamic Light Scattering and Native-Gel electrophoresis. The calibration of the gel filtration column (see "Materials & Methods") allowed us to evaluate the apparent molecular mass of the proteins according to their elution volume. TraB_FL _ran as a 198.1 kDa protein, TraB_NT _as a 122.1 kDa protein, TraB_CT _as a 100 kDa protein, and LvhB4 as a 92.7 kDa protein. By comparison with the calculated molecular mass obtained from the amino acid sequence, we concluded that TraB_FL_, TraB_NT _and TraB_CT _were all forming dimers under the examined buffer conditions. In contrast, LvhB4 behaved as a monomer in the same conditions. DLS and blue-native PAGE (BN-PAGE) confirmed these results for TraB_FL _and TraB_CT _(Table 1). In conclusion, all TraB-derived constructs form only dimers in solution, whereas LvhB4 forms monomers.

**Table 1:** Theoretical and experimental Molecular Mass (MM) determination

	MM^Calc^	GF calibration	D.L.S	BN-PAGE	Oligomeric state
**TraB_FL_**	103	198	184	146 - 242	Dimer
**TraB_NT_**	55	122	ND	ND	Dimer
**TraB_CT_**	49	100	100	66 - 146	Dimer
**LvhB4**	97	93	105	ND	Monomer

All MM are given in kDa. "MMCalc" is the MM calculated from the amino-acid sequence, "GF" stands for gel filtration, "DLS" stands for Dynamic Light Scattering, "BN-PAGE" stands for Blue Native-PAGE. The oligomeric state is deduced from the comparison between MM^Calc ^and the experimental MM as determined by the other approaches. "ND" stands for non-determined.

### Overall SAXS Parameters

**Figure 2:**
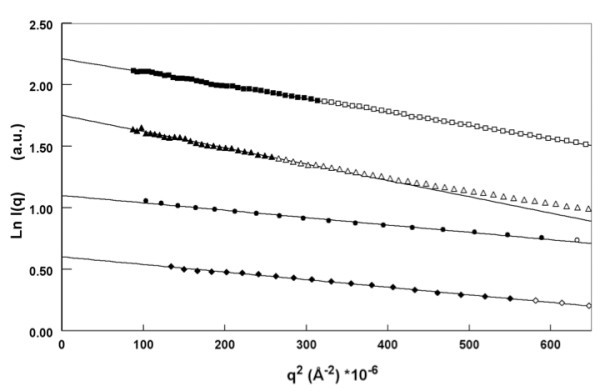
**Determination of the radius of gyration R_G _of the TraB constructs and of LvhB4 by the Guinier approximation**. The R_G _is inferred from the slope of the straight line fitting Ln I(*q*) vs *q*^2^, for *q*R_G _< 1.0. The points of the scattering curve used for the linear fit (straight line) are indicated by filled symbols, and the rest of the curve is represented by open symbols (TraB_FL_:square; Trab_NT_: triangle; LvhB4:circle; and TraB_CT_:diamond).

Small-angle X-ray scattering (SAXS) studies yield information on the size and shape of macromolecules in solution, and also on the oligomerisation state of macromolecules. The overall dimensions of a protein can be evaluated by its radius of gyration, *R*_G_, while the molecular mass of the scattering particle is inferred from the forward scattering intensity, *I*(0), both derived from the Guinier law (see Materials and Methods). The scattering curves of all the constructs followed the Guinier law very well (Figure 2) and did not display any sign of aggregation in solution. We determined the molecular mass of each construct of TraB from their experimental *I*(0) and obtained 219 kDa for TraB_FL_, 106 kDa for TraB_NT_, and 83 kDa for TraB_CT _(Table 2). After comparison with the expected molecular mass of the monomer of each TraB-derived constructs we concluded that all of them were dimers under the examined solution conditions (Table 2). For TraB_FL _and TraB_NT_, the molecular masses inferred from the forward scattering intensity are very similar to the theoretical molecular masses calculated from their sequence. This strongly suggests that very few detergent molecules are bound to the proteins, and therefore that the contribution of the detergents to the scattering intensity can be considered as negligible. LvhB4 was confirmed to be a monomer with a molecular mass experimentally calculated to be 116 kDa, very close to the expected monomer size of 97 kDa. The *I*(0) determination of the molecular mass of all four proteins were consistent with our biochemical results and confirmed their oligomeric states. These results suggest that both the N- and C-terminal halves of TraB participate in the dimer interface of the full-length protein.

**Table 2:** Biophysical parameters estimated by SAXS

Protein	MM_Calc _(kDa)	MM_Exp _(kDa)	O.S	*R*_G_* (Å)	*R*_G _** (Å)	*D*_max _(Å)	*R*_G_**/*D*_max_	*V *(Å^3^)	GASBOR ChiExp
TraB_FL_	103	219	2	58.6 ± 0.6	58.8	198 ± 2	0.30	2.4 × 10^5^	1.96
TraB_NT_	55	106	2	60.3 ± 0.7	59.2	195 ± 5	0.30	1.3 × 10^5^	1.56
TraB_CT_	49	83	2	41.5 ± 0.5	46.1	150 ± 5	0.31	1.1 × 10^5^	0.61
LvhB4	97	116	1	37.2 ± 0.6	38.1	120 ± 5	0.32	1.1 × 10^5^	2.01

(TraB_NT_+TraB_CT_)/TraB_FL_	1.01	0.86						1.0	
2(LvhB4)/TraB_FL_	0.94	1.06						0.92	

MM_Calc_: Theoretical molecular mass calculated from amino acid sequence. MM_Exp_: Experimentally based molecular mass calculated from the scattering intensity extrapolated at zero angles *I*(0). O.S: Oligomeric state, as determined by comparison between the MM_Calc _and the MM_Exp_. *R*_G_*: Radius of gyration, estimated from the Guinier plots. *R*_G_**: Radius of gyration, estimated using the program GNOM. *V*: volume of the envelope calculated by *ab initio *modelling. *ChiExp*: discrepancy between the experimental SAXS profile and the fit for each model calculated by the program GASBOR. The ratios of the Molecular Masses and of the volumes between different constructs are given in the last two lines.

The radii of gyration measured on the different constructs are summarized in Table 2. Surprisingly, TraB_FL _and TraB_NT _have similar radii of gyration (58.6 ± 0.6 Å and 60.3 ± 0.7 Å respectively), in spite of the molecular mass of TraB_NT _being half of that of TraB_FL_. In contrast, the radii of gyration of TraB_CT _and LvhB4 are smaller (41.5 ± 0.5 Å and 37.2 ± 0.6 Å respectively). Proteins of similar radius of gyration may have very different shape and mass depending on their structure. Thus, TraB_FL _and TraB_NT _may have similar R_G _values but different structures. According to the SAXS results, TraB_CT _and consequently TraB_FL _are on average more compact than TraB_NT_. The R_G _of TraB_NT _reflects a less compact structure with a lower molecular mass, whereas, the R_G _of TraB_FL _results from a more compact structure and higher molecular mass, these two parameters counter-balancing each other to yield similar R_G _values.

We then calculated the pair-distance distribution function, *P*(r), from the SAXS curves (see "Materials & Methods"). The *P*(r) functions for all four constructs exhibited a bell-shaped curve with a slightly extended profile for the higher distances (data not shown), indicating a globular but somewhat elongated conformation. The comparison of the values obtained for the radius of gyration (*R*_G_) and for the maximum dimension (*D*_max_) for the four proteins gives an idea of their anisotropy. To obtain an estimation of the anisotropy of the protein, we calculated the ratio between *R*_G _and *D*_max _values for each construct. In the case of a sphere, this ratio is equal to 0.39, as the radius of a sphere is equal to (3/5)^1/2^R, where R is the radius of the sphere. Table 2 summarizes the values computed for all the constructs. The ratio *R*_G_/*D*_max _is 0.30 for TraB_FL_, 0.30 for TraB_NT_, 0.31 for TraB_CT _and 0.32 for LvhB4, significantly different than the value for a sphere, considering the error bars measured on the R_G _and D_max_. Interestingly, despite having different sizes, all four constructs exhibit the same anisotropy (ratio *R*_G_/*D*_max _of ~0.3), indicating that the proteins are rather anisotropic, and thus elongated.

### Low Resolution Shapes from Ab Initio Modeling

**Figure 3:**
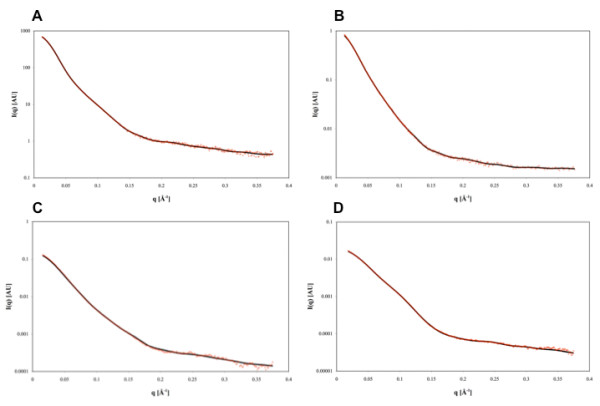
**Scattering curves of the different constructs of TraB and of LvhB4**. The calculated *I*(q) profiles (black line) of the four different structures restored from the SAXS data are compared with the measured SAXS data (red circles) for TraB_FL _(A); TraB_NT _(B); TraB_CT _(C) and LvhB4 (D). "AU" stands for "Arbitrary Units".

**Figure 4:**
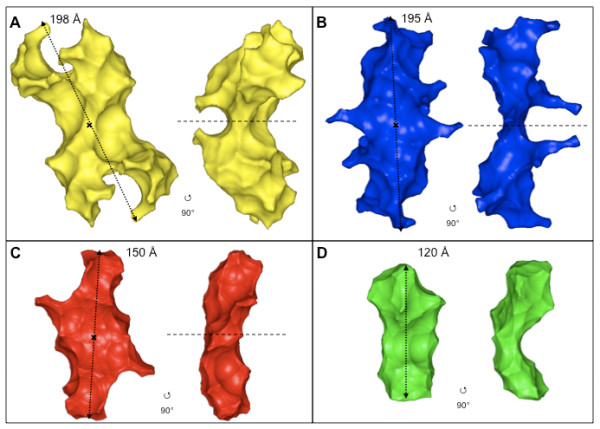
***Ab initio *models of the different constructs of TraB and of LvhB4**. Two orientations, rotated along the long axis, of the best models generated by the program GASBOR of TraB_FL _(A); TraB_NT _(B); TraB_CT _(C) and LvhB4 (D). All TraB models (FL; NT; CT) were generated with an imposed P2 symmetry. The black cross and the dashed-line indicate the P2 symmetry axis between the two monomers in the dimeric model. The double arrow-head dotted-line indicates the maximum dimension (*D*_max_) of each model.

The overall shapes of the four proteins were computed from the SAXS profiles using the program GASBOR [19]. For all TraB derived constructs (TraB_FL_; TraB_NT _and TraB_CT_), we used an imposed 2-fold symmetry axis (referred as P2 in GASBOR) for generating the reconstructed models, in agreement with the oligomerisation state inferred from the biochemical data and from the forward scattering intensity *I*(0). Similar shapes were also obtained without imposing any symmetry (referred as P1 in GASBOR, data not shown). For LvhB4, no symmetry was imposed to reconstruct the 3D-volume. Several independent calculations provided highly reproducible results, with very similar models, and fit to the data of similar quality. The average shapes calculated from repeated, multiple modelling processes (data not shown) give each time a shape similar to the best individual model, defined by the lowest ChiExp value. Figure 3 shows that the fit to the experimental data of these shapes for all four proteins is very good, as confirmed by the value of ChiExp (Table 2). The very reproducible shapes together with the very low ChiExp, obtained when fitting the data (Table 2) give good confidence that the inferred model is not an artifact due to the SAXS intrinsic degeneracy and that these shapes are reliable. We decided to use the best model for each construct as representative of their low resolution structure in solution. Overall, the shapes of the four proteins appear to be globular but rather elongated (Figure 4), as expected from the *R*_G_/*D*_max _ratio. TraB_FL _presents two symmetrical lobes with two distinctive protruding ends, with each lobe being potentially attributable to one TraB_FL _monomer in the dimeric assembly (Figure 4A). On the other hand, the shapes of both TraB_NT _(Figure 4B) and TraB_CT _(Figure 4C) appeared to be rather flat and elongated with few protruding extensions. The symmetrical 2-fold axis separating the two monomers is however clearly visible in the reconstructed volumes, even without imposing any symmetry (data not shown). Interestingly, the low-resolution structure of LvhB4 (Figure 4D) shows an asymmetrical and compact, yet rather extended conformation.

### Superposition and comparison of the TraB derived models

**Figure 5:**
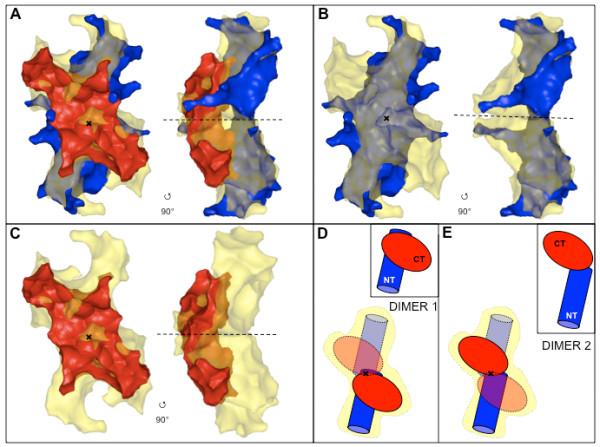
**Assembly of TraB-domain SAXS models, reconstituted with the program GASBOR**. (A) The TraB_NT _(blue) and TraB_CT _(red) models are superimposed onto the TraB_FL _model (transparent yellow). (B) The TraB_NT _model is superimposed onto the TraB_FL _model. (C) The TraB_CT _model is superimposed onto the TraB_FL _model. (D and E) Cartoons representing the domain organisation of TraB deduced from the SAXS models. Two possibilities for the TraB_FL _monomer are shown, with dimer 1 (D) and dimer 2 (E). The inset shows the TraB_FL _monomer. In the dimer, one monomer is represented in plain lines without transparency, the other monomer is represented in dotted-lines and transparency. The black cross and the dashed-line both indicate the P2 symmetry axis of the dimeric models. The SAXS models are shown in two orientations, with a 90° degrees angle rotation along the long axis.

TraB_NT _and TraB_CT _represent the N- and C-terminal halves of TraB_FL_, respectively (see Figure 1 and "Materials & Methods"). Thus, given that all the three TraB-derived constructs are dimeric, the sum of the reconstructed volumes of both TraB_NT _and TraB_CT _should give a value similar to the volume of TraB_FL_. We used the program CRYSOL [20] to evaluate the volume of the reconstructed models (Table 2). We found that the sum of the volumes of TraB_NT _and TraB_CT _gives a value very close to the volume of TraB_FL _(represented here by the ratio of the volumes being close to 1.0, see table 2). We then manually superimposed the shapes of TraB_NT _and TraB_CT _onto the shape of TraB_FL_. Several respective orientations of TraB_NT _and TraB_CT _were tested and for only one orientation the TraB_NT _and TraB_CT _models fit well together into the TraB_FL _model without any clash (Figure 5A). In the proposed TraB_FL _model, the longest dimension of TraB_NT _and TraB_CT _are 45° apart, with the longest dimension of TraB_NT _coinciding with that of the TraB_FL _dimer. In this model, the more compact structure of TraB_CT _lays onto the more elongated structure of TraB_NT _(Figure 5B and 5C). Two schemes for the location of the TraB monomer and its two domains can be inferred from these SAXS envelopes of TraB_FL _and TraB fragments (Figure 5D and 5E). In one scheme (dimer 1, Figure 5D), each TraB monomer is positioned on either side of an axis perpendicular to the long axis of the TraB dimer. In the other scheme (dimer 2, Figure 5E), each TraB monomer is positioned on each side of the long TraB dimer axis.

### Comparison between TraB_FL _and LvhB4 and orientation of the TraB monomers

**Figure 6:**
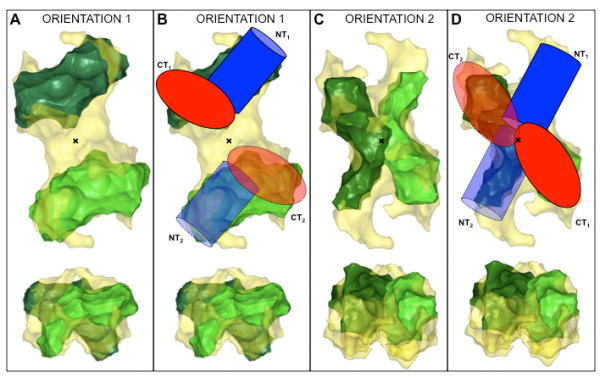
**Comparison between TraB_FL _model and LvhB4 model reconstituted from SAXS data**. Two possible orientations of two LvhB4 monomers into the TraB_FL _model, with (A, ORIENTATION 1)) the two LvhB4 monomers are aligned along the shortest axis of the TraB_FL _model, or (C, ORIENTATION 2) the two LvhB4 monomers (in light- and dark-green) are aligned along the longest axis of the TraB_FL _model (in transparent yellow). (B): same as A but for the cartoon representation of TraB in Figure 5D being superimposed. (D): same as C but for the cartoon representation of TraB in Figure 5E being superimposed. Each panel shows two perpendicular views of the model. The two LvhB4 monomers were manually fitted into the TraB_FL _model. The black cross and the dashed-line both indicate the P2 symmetry axis of the dimeric models.

VirB4 proteins are the most conserved components (amino-acid sequence wise) of the T4S systems. It was then logical to think that VirB4 proteins could have a similar shape, albeit with different oligomeric states. We thus hypothesised that if TraB_FL _and LvhB4 monomers (34% identity) share a similar overall tertiary structure, the latter could help us localise TraB_FL _monomers in the dimeric model. The ratio between the volumes of two LvhB4 monomers and one dimeric TraB_FL _is of the same order as the ratio of their molecular masses, with LvhB4 slightly smaller than TraB_FL _monomers (Table 2). Therefore, we tried to manually fit two LvhB4 monomers into the envelope of the TraB_FL _dimer. Figure 6 shows the results obtained with two possible orientations: orientation 1 (Figure 6A and 6B) with each of the LvhB4 monomer being on each side of an axis perpendicular to the TraB_FL _dimer longest axis; and orientation 2 (Figure 6C and 6D) with each LvhB4 monomer being on each side of the longest axis of the TraB_FL _dimer. In Figure 6B and 6D, we have superimposed the schematic diagrams of Figure 5D and 5E, respectively. In both orientations, there remains empty spaces in the TraB_FL _dimer, not filled by two LvhB4 monomers. In the case of orientation 1, this empty space is localized in between the two monomers. This is not in agreement with the evidence of a stable TraB_FL _dimer and with the fact that both the N- and C-terminal domains have been also isolated as dimers. Conversely, in the case of orientation 2, this empty space is localized at the N-terminal ends of LvhB4. This is consistent with the shorter N-terminal domain of LvhB4 compared to TraB and subsequently with the smaller volume of the LvhB4 model compared to the corresponding volume of the TraB_FL _monomer (taken as half of the observed TraB_FL _dimer volume; Table 2). In addition, the contact areas between the two N-terminal domains and the two C-terminal domains are larger, allowing formation of stable TraB_FL_, TraB_NT _and TraB_CT _dimers (see cartoons in Figure 6D). In conclusion, we favor orientation 2 as best describing the possible arrangement of the TraB_FL _monomers into the dimeric model.

### Comparison between TraB_CT _model and the homology-based structure of At-VirB4

**Figure 7:**
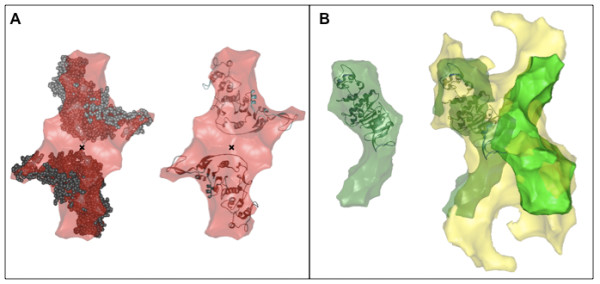
**Localisation of the C-terminal domain of *At*-VirD4, in TraB_CT _and in LvhB4**. (A) SAXS model of TraB_CT _dimer (transparent red surface) superimposed with the *At*-VirB4-Cter atomic model (in ribbons and CPK representations). The black cross indicates the two-fold symmetry axis of the dimeric TraB_CT _model. (B) SAXS model of LvhB4 monomer (transparent green surface) superimposed with the *At*-VirB4-Cter atomic model (in ribbons representation); two SAXS models of LvhB4 monomer were fitted into the TraB_FL _SAXS model (transparent yellow surface). The *At*-VirB4-Cter monomers were manually fitted into the TraB_CT _and LvhB4 models.

As mentioned previously, Middleton *et al. *[17] have modeled the structure of the C-terminal domain of the *A. tumefaciens *VirB4 (*At*-VirB4-Cter) based on the sequence homology with the VirD4 protein TrwB from the R388 conjugation machinery. Since TraB and *At*-VirB4 are highly homologous, we used this *At*-VirB4-Cter model and tried to fit two of them into the TraB_CT _dimeric shape. Indeed, as shown in Figure 7 (panel A), we could manually fit the two *At*-VirB4-Cter models into the TraB_CT _dimer volume. The two *At*-VirB4-Cter subunits are arranged according to the two-fold symmetry axis of the TraB_CT _dimer. As shown in Figure 7A, this docking identifies the C-terminal end of TraB as participating in the dimer interface. Similarly, we superimposed the *At-*VirB4-Cter model with the SAXS envelope of LvhB4 (Figure 7, panel B). From this comparison we propose that the C-terminal domain of LvhB4 is possibly localised at the wider end of the curved shape, opposite to the narrow end. The empty space in the superimposition would consequently be the N-terminal domain of LvhB4. This orientation fits well with the models presented in Figure 6C and 6D, showing the superimposition of two LvhB4 monomers into the TraB_FL _dimeric model.

## Discussion

The SAXS experiments reported here confirm that TraB and its N- and C-terminal domains are dimeric in the acetate-free solution conditions under which the experiments were conducted, indicating that both domains participate in the dimer interface. The structures of the two domains revealed elongated shapes, which in the full-length protein come together at a 45° angle. The superposition of the LvhB4 structure could resolve the ambiguity as to where the TraB monomer lies, and favored dimer 2 (Figure 5E), where the monomer would extend along the long axis of the dimer structure. Indeed, in the superimpositions of the two LvhB4 monomers onto the TraB_FL _dimer structure presented in Figure 6B and 6D, only the one aligning the LvhB4 monomers along the long axis of the TraB structure gives rise to an extended dimer interface. The configuration in Figure 5D (Dimer 1) would be expected to yield a less stable dimer than is observed. Also, a more extended conformation of TraB is consistent with our observation that TraB is susceptible to proteolysis and that limited proteolysis of TraB very rapidly yields TraB_CT _(data not shown).

VirB4 is a family of very conserved proteins that are essential components of T4S systems [12] However, recent biochemical studies have revealed that this family of proteins is more diverse than originally expected [13]. For example, their oligomerisation state appears different depending on the system under investigation and the conditions under which they are studied. TraB has been shown to be in equilibrium between two oligomeric states, dimer and hexamer, dependent on the solution conditions, namely the presence or absence of acetate ion [15]. TrwK appears to transition between a major monomeric form and minor hexameric form [14]. The VirB4 homolog encoded by the *cag *pathogenicity island in *H. pylori *appears to be monomeric [13] and we show here that, under the solution conditions examined, the VirB4 homolog from *L. pneumophila *LvhB4 is monomeric. Hexamer formation appears to be required for ATP-hydrolyzing activity: indeed only hexameric forms of VirB4 homologs have been shown to exhibit ATPase activity [14,15]. So far, only sparse information has been gathered about the function of the dimeric TraB. We recently reported its DNA and nucleotide binding activities [15], while *A. tumefaciens *VirB4 was shown to direct dimer formation when fused to the N-terminal portion of the cI repressor protein [16]. The different subcellular localisation of TraB together with the recent characterization of a degenerated nucleotide binding site in its N-terminal domain [15] are features also observed in the SecA translocase, perhaps suggesting an evolutionary relationship between the two protein families [21,22]. Finally, TraB in the context of the entire T4S machinery might interact with different partners. Indeed, its close association with TraA (the VirB3 homolog encoded by the pKM101 plasmid) or its documented protein-protein interactions with other T4S system components could induce the conformational changes necessary to reshape the active site or radically change its cell environment more specifically, in order to stabilise an active dimeric membrane-bound form.

## Conclusions

The work presented here provides the first structural glimpse of a protein which is crucial to type IV secretion but has until now resisted X-ray crystallography or EM structural characterisation. It uncovers a modular structure that comes together in an extended dimer interface where the domains appear to "hug" each other. The dimers corresponding to each domain could easily be put together in the envelope of the full length protein and the structural model for LvhB4 helps suggest a potential model for the full-length protein. Intriguingly, the predicted TM segment locates within the N-terminal domain, not at the boundaries of the domain structure. This raises topological issues that can be resolved by a model invoking an orientation of the TraB_NT _domain facing the cytosolic side of the inner membrane, while the TraB_CT _domain would lie in the cytoplasm. This would be consistent with TraB being only superficially associated with the membrane, and therefore being able to partition between the membrane and the cytoplasm. It is also consistent with the dimeric structure proposed here. Docking of the TrwB protein (a potential structural homolog of the C-terminal domain of VirB4 proteins) within the envelope of the TraB_CT _provides further structural details. Finally, the dimeric model of TraB observed here suggests that there might be structural rearrangements required to fit the VirB4 dimeric structure into the 14-fold symmetrical core complex recently unravelled by the high resolution EM structure of the VirB7-VirB9-VirB10 complex [23] and confirmed by the subsequent crystal structure of its outer membrane-inserting part [24]. Further studies will seek to elucidate the crystal structure of a VirB4 protein and also to visualize a complex of VirB4 bound to the core complex.

## Methods

### Cloning of TraB domains and LvhB4

Cloning of the full-length *tra*B gene (*tra*B_FL_, amino acids 1-866; Figure 1), the region encoding the N-terminal domain (*tra*B_NT_, amino acids 1-442; Figure 1) and the C-terminal domain (*tra*B_CT_, amino acids 448-848; Figure 1), together with the full-length *lvh*B4 gene (*lvh*B4 amino acids 1-826; *Legionella pneumophila *strain JR32) was as described in Durand *et al. *[15]. All four constructs allow the expression of N-terminally His_6_-tagged recombinant proteins, referred to thereafter as TraB_FL_, TraB_NT_, TraB_CT_, or LvhB4. After DNA sequencing (MWG Biotech) to check that the sequences did not contained any mutation, the four plasmids were transformed by heat-shock in chemically competent BL21 star (DE3) cells (Invitrogen), for large scale production of the recombinant proteins.

### Production and Purification of Recombinant Proteins

*E. coli *strain BL21 star/DE3 (Invitrogen) containing one of the recombinant plasmids was grown at 37°C in Terrific Broth supplemented with 100 μg/ml of Ampicillin (Sigma-Aldrich), until the culture reached an A_600 nm _of 1.2. Cultures were then shifted to 16°C for 1 h, before isopropyl-β-D-thiogalactopyranoside (IPTG) was added to a final concentration of 1 mM and growth continued for 15 h at 16°C. Cells were harvested by centrifugation, resuspended in 20 mM TrisHCl (pH7.5) and store at -20°C.

All subsequent steps were carried out at 4°C. TraB_CT _and LvhB4 were purified from cytoplasmic extracts as follow. The cells were defrosted and one tablet of Protease inhibitor cocktail EDTA free (Roche) was added, together with 300 mM NaCl and 1 mM β-mercapto-ethanol (βME). After cells were broken by two rounds through an EmulsiFlex-C5 homogeniser and DNA fragmentation by sonication, the lysate was clarified by centrifugation at 18,000 r.p.m. for 45 min in a Sorvall SS-34 rotor. The clarified lysate was loaded onto a HisTrapHP 5 ml column (GE Healthcare) equilibrated in buffer A^sol ^(20 mM TrisHCl/pH7.5, 300 mM NaCl, 1 mM βME) plus 4% of buffer B^sol ^(20 mM TrisHCl/pH7.5, 300 mM NaCl, 1 mM βME, 500 mM Imidazole). The column was then washed with 100 ml of buffer A^sol ^plus 8% buffer B^sol^. Finally the proteins still bound to the column were eluted in a gradient from 4% to 100% of buffer B^sol ^in 100 ml. Eluted fractions containing either TraB_CT _or LvhB4 were pooled and concentrated in less than 4 ml before being loaded onto a HiPrep 16/60 Sephacryl S-300 HR column (Amersham) equilibrated in buffer GF^sol ^(20 mM Tris/HCl pH 7.5, 50 mM NaCl, 1 mM βME). The proteins TraB_CT _and LvhB4 both eluted as a single peak. Fractions under this peak were pooled.

TraB_FL _and TraB_NT _were purified from membrane extracts as followed. The cells were defrosted and one tablet of protease inhibitor cocktail EDTA free (Roche) was added, together with 50 mM NaCl and 1 mM βME. After cells were broken by two rounds through an EmulsiFlex-C5 homogeniser and DNA fragmentation by sonication, unbroken cells were removed by centrifugation at 14,000 r.p.m. for 10 min in a Sorvall SS-34 rotor. Total membranes were pelleted by ultracentrifugation (45 min at 100,000 g, 4°C) and resuspended in buffer EB (20 mM Tris-HCl/pH 7.5, 50 mM NaCl, 1 mM βME, 1% (v/v) Triton^® ^X-100) supplemented with one tablet of protease inhibitor cocktail EDTA free (Roche). Membrane-embedded proteins were extracted during 1 h at 4°C. The membrane extract was further clarified by ultracentrifugation (30 min at 100 000 g, 4°C). Triton^® ^X-100 was only used for extraction, then it was replaced by the hydrogenated Triton^® ^X-100^(H) ^(Calbiochem) that does not absorb in UV. We further used a concentration of 0.01% Triton^® ^X-100^(H) ^(0.16 mM) since it was below the CMC of the detergent (0.2-0.9 mM), thus avoiding the formation of detergent micelles. The cleared extract was loaded onto a HisTrapHP 5 ml column (GE Healthcare) equilibrated in buffer A^mb ^(20 mM TrisHCl/pH7.5, 300 mM NaCl, 1 mM βME, 0.01% Triton^® ^X-100^(H)^) plus 4% of buffer B^mb ^(20 mM TrisHCl/pH7.5, 300 mM NaCl, 1 mM βME, 0.01% Triton^® ^X-100^(H)^, 500 mM Imidazole). The column was then washed with 100 ml of buffer A^mb ^plus 8% buffer B^mb^. Finally the proteins still bound to the column were eluted in a gradient from 4% to 100% of buffer B^mb ^in 100 ml. Eluted fractions containing either ^His^TraB_FL _or ^His^TraB_NT _were pooled and concentrated in less than 4 ml before being loaded onto a HiPrep 16/60 Sephacryl S-300 HR column (Amersham) equilibrated in buffer GF^mb ^(20 mM Tris/HCl pH 7.5, 50 mM NaCl, 1 mM βME, 0.01% Triton^® ^X-100^(H)^). The proteins TraB_FL _and TraB_NT _both eluted as a single peak. Fractions under this peak were pooled. Apparent molecular mass of proteins eluted from the gel filtration column was deduced from a calibration carried out with low and high molecular mass calibration kits (Amersham Biosciences). Determination of protein concentration was carried out by either using the theoretical absorption coefficients at 280 nm, which were obtained using the program ProtParam on the EXPASY server (available on the World Wide Web at http://www.expasy.ch/tools), or with the Bio-Rad protein assay reagent (Bio-Rad).

### Dynamic Light Scattering (DLS)

Dynamic light scattering experiments were performed with a DynaPro-801 (Protein Solutions) at room temperature. All samples were filtered prior to the measurements (Millex syringe filters, 0.22 μm; Millipore Corp.). Diffusion coefficients were inferred from the analysis of the decay of the scattered intensity autocorrelation function. The hydrodynamic radius and the molecular mass (MM) of proteins in solution were both deduced from translational diffusion coefficients. All calculations were performed using the software provided by the manufacturer (Dynamics V5.25.44).

### SAXS Experiments

SAXS experiments were performed in two different locations. TraB_FL _and TraB_NT _were analysed on beamline X33 [25] at EMBL-Hamburg on storage ring DORIS III of the Deutsches Elektronen Synchrotron (DESY) using a MAR 345 image plate detector. The scattering patterns from solutions of TraB_FL _at protein concentrations of 3, 5, 7.5, 10, and 13.5 mg/ml, and for TraB_NT _at protein concentrations of 1.3, 2.1, 4.9, and 8.2 mg/ml were measured in buffer GF^mb^. At a sample detector distance of 2.7 m and wavelength (*λ*) of 1.5 Å, the scattering vectors, *q *ranging from 0.0093 Å^-1 ^to 0.50 Å^-1 ^was covered (*q = 4πsinθ/λ*, where 2*θ *is the scattering angle). According to radiation damage tests, one frame of 2 min exposure time was recorded for every sample. The data were normalised to the intensity of the transmitted beam and radially averaged, and the scattering of the buffer was subtracted, as absolutely no trace of the presence of micelles was detected from the buffer scattering curve. The difference curves were scaled for protein concentration and extrapolated to yield the final composite scattering curves. Molecular mass calibration was made with BSA.

TraB_CT _and LvhB4 were analysed at the European Synchrotron Radiation Facility (Grenoble, France) on beamline ID02 as described previously [26]. The scattering patterns from solutions of TraB_CT _at protein concentrations of 2.1, 3.7, 4.3, 6.1, and 8.2 mg/ml, and for LvhB4 at protein concentrations of 2, 2.9, 4.6, 6, 7.3, and 8.9 mg/ml were measured in buffer GF^sol^. The wavelength was 1.0 Å. The sample-to-detector distances were set at 1.0 m (TraB_CT_) and 1.5 m (LvhB4), resulting in scattering vectors, *q *ranging from 0.011Å^-1 ^to 0.50 Å^-1 ^and from 0.010Å^-1 ^to 0.37 Å^-1 ^respectively. All experiments were performed at 20°C. Absolute calibration was made with water.

### SAXS Data Evaluation

All steps for data processing were performed using the program package PRIMUS [27]. The experimental SAXS data for all samples were linear in a Guinier plot of the low *q *region, indicating that the proteins did not undergo aggregation. The radius of gyration *R_G _*was derived by the Guinier approximation *I(q) = I(0) exp(-q^2^R_G_^2^/3) *for *qR_G _*< 1.0. The radii of gyration *R_G_*, calculated for different protein concentrations, displayed a slight concentration dependence arising from particle interferences in solution. Interference-free SAXS profiles were estimated by extrapolating the measured scattering curves to infinite dilution. The molecular masses of the solutes were inferred from *I*(0) values, the forward scattering intensity, which is proportional to the molecular mass of the protein according to relationship *MM ~I(0)/c*, where c is the protein concentration. The intensity *I(0) *was experimentally inferred from the intercept of the linear fit in the Guinier plot *Ln[I(q)] versus q^2 ^*at low *q *values (*qR_G _*< 1.0). The program GNOM [28] was used to compute the pair-distance distribution functions, *P(r)*. This approach also features the maximum dimension of the macromolecule, Dmax.

### Ab Initio Modeling

The overall shapes of the entire assemblies were restored from the experimental data using the program GASBOR [19]. The scattering profiles were fitted on the spectrum of each protein up to q = 0.37Å^-1^. GASBOR searches a chain-compatible spatial distribution of an exact number of dummy residues, centred on the C_α _atoms of the protein amino acid residues. We used both symmetry operations P1 and P2 proposed by the program GASBOR. At least 10 low resolution models obtained from different runs were averaged using the program DAMAVER [29] to construct the average model representing the general structural features of each reconstruction.

## Authors' contributions

VRB and GW designed the study and finalised the manuscript, and ED realised the experiments, analysed the SAXS data with VRB, and drafted the manuscript. All authors read and approved the final manuscript.
